# Nrg4 promotes fuel oxidation and a healthy adipokine profile to ameliorate diet-induced metabolic disorders

**DOI:** 10.1016/j.molmet.2017.03.016

**Published:** 2017-06-21

**Authors:** Zhimin Chen, Guo-Xiao Wang, Sara L. Ma, Dae Young Jung, Hyekyung Ha, Tariq Altamimi, Xu-Yun Zhao, Liang Guo, Peng Zhang, Chun-Rui Hu, Ji-Xin Cheng, Gary D. Lopaschuk, Jason K. Kim, Jiandie D. Lin

**Affiliations:** 1 Life Sciences Institute and Department of Cell & Developmental Biology, University of Michigan Medical Center, Ann Arbor, MI 48109, USA; 2 Program in Molecular Medicine and Division of Endocrinology, Metabolism and Diabetes, Department of Medicine, University of Massachusetts Medical School, Worcester, MA 01605, USA; 3 423 Heritage Medical Research Building, University of Alberta Edmonton, Alberta T6G 2S2, Canada; 4 Weldon School of Biomedical Engineering and Department of Chemistry, Purdue University, West Lafayette, IN 47907, USA

**Keywords:** Adipose tissue, Brown fat, Nrg4, Adipokine, NAFLD, Diabetes, BAT, Brown adipose tissue, WAT, White adipose tissue, eWAT, epididymal WAT, Nrg4, Neuregulin 4, HFD, High-fat diet, TAG, Triglyceride, WT, Wild type, Tg, Transgenic, KO, Knockout, TNFα, Tumor necrosis factor α, NALFD, Non-alcoholic fatty liver disease, VEGFα, Vascular endothelial growth factor α, FGF21, Fibroblast growth factor 21, IL-6, Interleukin-6, BMPs, Bone morphogenetic proteins, UCP-1, Uncoupling protein 1, GPR120, G-protein coupled receptor 120, CoA, Co-enzyme A

## Abstract

**Objective:**

Brown and white adipose tissue exerts pleiotropic effects on systemic energy metabolism in part by releasing endocrine factors. Neuregulin 4 (Nrg4) was recently identified as a brown fat-enriched secreted factor that ameliorates diet-induced metabolic disorders, including insulin resistance and hepatic steatosis. However, the physiological mechanisms through which Nrg4 regulates energy balance and glucose and lipid metabolism remain incompletely understood. The aims of the current study were: i) to investigate the regulation of adipose Nrg4 expression during obesity and the physiological signals involved, ii) to elucidate the mechanisms underlying Nrg4 regulation of energy balance and glucose and lipid metabolism, and iii) to explore whether Nrg4 regulates adipose tissue secretome gene expression and adipokine secretion.

**Methods:**

We examined the correlation of adipose Nrg4 expression with obesity in a cohort of diet-induced obese mice and investigated the upstream signals that regulate Nrg4 expression. We performed metabolic cage and hyperinsulinemic-euglycemic clamp studies in Nrg4 transgenic mice to dissect the metabolic pathways regulated by Nrg4. We investigated how Nrg4 regulates hepatic lipid metabolism in the fasting state and explored the effects of Nrg4 on adipose tissue gene expression, particularly those encoding secreted factors.

**Results:**

Adipose Nrg4 expression is inversely correlated with adiposity and regulated by pro-inflammatory and anti-inflammatory signaling. Transgenic expression of Nrg4 increases energy expenditure and augments whole body glucose metabolism. Nrg4 protects mice from diet-induced hepatic steatosis in part through activation of hepatic fatty acid oxidation and ketogenesis. Finally, Nrg4 promotes a healthy adipokine profile during obesity.

**Conclusions:**

Nrg4 exerts pleiotropic beneficial effects on energy balance and glucose and lipid metabolism to ameliorate obesity-associated metabolic disorders. Biologic therapeutics based on Nrg4 may improve both type 2 diabetes and non-alcoholic fatty liver disease (NAFLD) in patients.

## Highlights

• Nrg4 is a target of pro-inflammatory and anti-inflammatory signaling in adipocytes.
• Transgenic expression of Nrg4 increased energy expenditure and glucose metabolism.
• Nrg4 stimulates hepatic fatty acid oxidation and ketogenesis during starvation.
• Nrg4 promotes a beneficial adipokine profile during obesity.

## 1 Introduction

Adipose tissue is central to systemic energy balance and metabolic homeostasis. White adipose tissue (WAT) stores lipids and communicates with the central nervous system and other peripheral tissues by secreting a number of important endocrine hormones [1], [2], [3]. WAT also serves as a major site for the integration of immune and metabolic signals [4], [5], [6]. The primary role of brown adipose tissue (BAT) is fuel oxidation linked to thermogenesis, which serves an important function in defense against cold and energy excess [7], [8], [9]. Secreted factors are important regulators of carbohydrate and lipid metabolism and systemic energy homeostasis. Adipose tissue hormones, such as leptin and adiponectin [1], [2], [3], gut-derived fibroblast growth factors [10], [11], and myokines [12] participate in nutrient sensing and coordinate key aspects of nutrient and energy metabolism. By integrating mouse tissue and brown adipogenesis transcriptome data, a list of brown fat-enriched secreted factors was identified [13]. Among these, Neuregulin 4 (Nrg4) emerged as a BAT-enriched endocrine factor that exerts robust effects on hepatic lipid metabolism and systemic homeostasis. Brown fat is also known to release factors that exhibit a wider expression profile, such as Vascular endothelial growth factor α (VEGFα), Fibroblast growth factor 21 (FGF21), Interleukin-6 (IL-6), and Bone morphogenetic proteins (BMPs) [14], [15]. As such, brown fat is emerging as a source of endocrine factors that exerts powerful metabolic effects beyond UCP1-mediated thermogenesis [14], [15].

Nrg4 expression is more abundant in brown fat than white fat; a similar pattern was observed in a recent study on differential gene expression in adipose tissue [16]. Despite its enrichment in brown fat, Nrg4 expression is readily detectable in white fat, which likely provides a significant source of total Nrg4 in circulation given its relatively large mass. An intriguing aspect of Nrg4 regulation is that its expression in WAT is markedly downregulated in mouse and human obesity [13]. These findings raise the possibility that obesity is associated with a functional deficit of Nrg4 that exacerbates the progression of metabolic disorders. In support of this, mice lacking Nrg4 developed more severe insulin resistance and hepatic steatosis following high-fat feeding, whereas fat-specific transgenic expression of Nrg4 significantly improves metabolic parameters and ameliorates diet-induced disruption of homeostasis. At the molecular level, Nrg4 transduces signals through the ErbB4 and ErbB3 receptor tyrosine kinases and elicits an inhibitory effect on hepatic lipogenesis. Despite these intriguing findings, the physiological mechanisms through which Nrg4 modulates energy balance and glucose and lipid metabolism remain incompletely understood. In this study, we performed metabolic cage and hyperinsulinemic-euglycemic clamp studies and revealed a surprisingly pleiotropic effect of Nrg4 signaling on key aspects of systemic energy and glucose metabolism. We identified hepatic fatty acid oxidation and ketogenesis as a new metabolic target of endocrine signaling by Nrg4. Finally, we found that Nrg4 promotes a beneficial adipokine profile during obesity. Together, this work provides important insights into the physiological actions of Nrg4 that contribute to its beneficial effects on metabolic homeostasis.

## 2 Materials and methods

### 2.1 Mouse studies

All mouse studies were performed according to procedures approved by the University Committee on Use and Care of Animals at the University of Michigan. The generation of Nrg4 transgenic and knockout mice was described previously [13]. Mice were maintained under 12/12 h light/dark cycles with free access to food and water. Teklad 5001 laboratory diet was used as a standard chow. For HFD feeding, mice were fed a diet containing 60% of calories from fat (D12492, Research Diets). Metabolic cage study was performed at the University of Michigan Animal Phenotyping Core using CLAMS (Columbus Instruments). The measurements were carried out for a total of 72 h. Energy expenditure was calculated as the value corresponding to 3.91VO_2_ + 1.1VCO_2_. Body fat and lean mass were measured using an NMR analyzer (Minispec LF90II, Bruker Optics). Plasma concentrations of TAG, total cholesterol, NEFA, and β-hydroxybutyrate were measured using commercial assay kits (Stanbio Laboratory). Plasma insulin and leptin concentrations were measured using ELISA (Crystal Chem).

### 2.2 Hyperinsulinemic-euglycemic clamp

Mice were fasted overnight (approximately 15 h), and a 2-h hyperinsulinemic-euglycemic clamp was conducted in awake mice with a primed and continuous infusion of human insulin (150 mU/kg body weight priming followed by 2.5 mU/kg/min; Novolin, Novo Nordisk, Plainsboro, NJ) [17]. To maintain euglycemia, 20% glucose was infused at variable rates during clamps. Whole body glucose turnover was assessed with a continuous infusion of [3-^3^H]glucose (PerkinElmer, Waltham, MA), and 2-deoxy-D-[1-^14^C]glucose (2-[^14^C]DG) was administered as a bolus (10 μCi) at 75 min after the start of clamps to measure insulin-stimulated glucose uptake in individual organs. At the end of the clamps, mice were anesthetized, and tissues were taken for biochemical analysis as previously described [17].

### 2.3 Adipocyte studies

3T3-L1 preadipocytes were cultured in DMEM with 10% bovine growth serum until two days post confluence (denoted as day 0). Differentiation was induced by adding a cocktail containing 0.5 mM IBMX, 1 μM dexamethasone, and 1 μg/mL insulin to DMEM supplemented with 10% FBS. Three days after induction, cells were cultured in DMEM containing 10% FBS plus 1 μg/mL of insulin for two more days followed by maintenance in DMEM supplemented with 10% FBS. Differentiated adipocytes were treated with TNFα, VIII compound, Rosiglitazone, GW9508 and DHA for 17 h in maintenance media before RNA isolation and RT-qPCR analysis.

### 2.4 qPCR and immunoblotting analyses

RT-qPCR analysis of gene expression was performed as previously described [18]. Briefly, total adipocyte and liver RNA was extracted using TRIzol method. Total RNA from eWAT and BAT was isolated using PureLink RNA isolation kit (ThermoFisher). For RT-qPCR, 2 μg of total RNA was reverse-transcribed using MMLV-RT followed by qPCR using SYBR Green (Life Technologies). Relative mRNA expression was normalized to the levels of ribosomal protein 36B4. Total eWAT lysates were prepared by tissue homogenization in a lysis buffer containing 50 mM Tris (pH 7.5), 150 mM NaCl, 5 mM NaF, 25 mM β-glycerolphosphate, 1 mM sodium orthovanadate, 10% glycerol, 1% tritonX-100, 1 mM dithiothreitol, and freshly added protease inhibitors. Immunoblotting was performed using specific antibodies against adipsin (Santa Cruz Biotech, sc-50419).

### 2.5 Microarray analysis

Gene expression profiling was performed using Mouse Gene ST 2.1 array. We used a cutoff of normalized array values (log2-transformed values > 7.0) for adipose tissue transcripts. Pathway enrichment analysis was performed using the Database for Annotation, Visualization and Integrated Discovery (DAVID, available at http://david.abcc.ncifcrf.gov). Adipose tissue gene expression was visualized using TreeView.

### 2.6 Stimulated Raman scattering (SRS) microscopy

SRS microscopy was used to evaluate lipid accumulation in hepatocytes as previously described [19]. Briefly, liver specimens were sectioned into a series of 50 μm thickness tissue slices by a vibratome. Single slice was sandwiched between two coverslips for SRS imaging of lipids with the beating frequency tuned to 2850 cm^−1^.

### 2.7 Hepatic lipid and CoA analyses

Hepatic lipids were extracted using Bligh-Dyer method in the presence of an internal standard (T21:0 TAG, 10 nmol/mg protein) and separated on silica gel 60 Å plates that were developed with a nonpolar acidic mobile phase (70:30:1, v/v/v, hexane/ethyl ether/acetic acid). Fatty acid profiling was performed using procedure described previously [13]. CoA ester levels were assessed in perchloric acid extracted heart tissue using a high-performance liquid chromatography procedure described previously [20].

### 2.8 Statistical analysis

Statistical analysis was performed using GraphPad Prism 7. Statistical differences were evaluated using two-tailed unpaired Student's *t*-test or one-way analysis of variance (ANOVA) with appropriate *post hoc* analyses. A p value of less than 0.05 (*p < 0.05, **p < 0.01, and ***p < 0.001) was considered statistically significant. Statistical methods and corresponding p values for data shown in each panel were included in figure legends.

## 3 Results

### 3.1 Regulation of adipose Nrg4 expression in obesity

**Figure 1:**
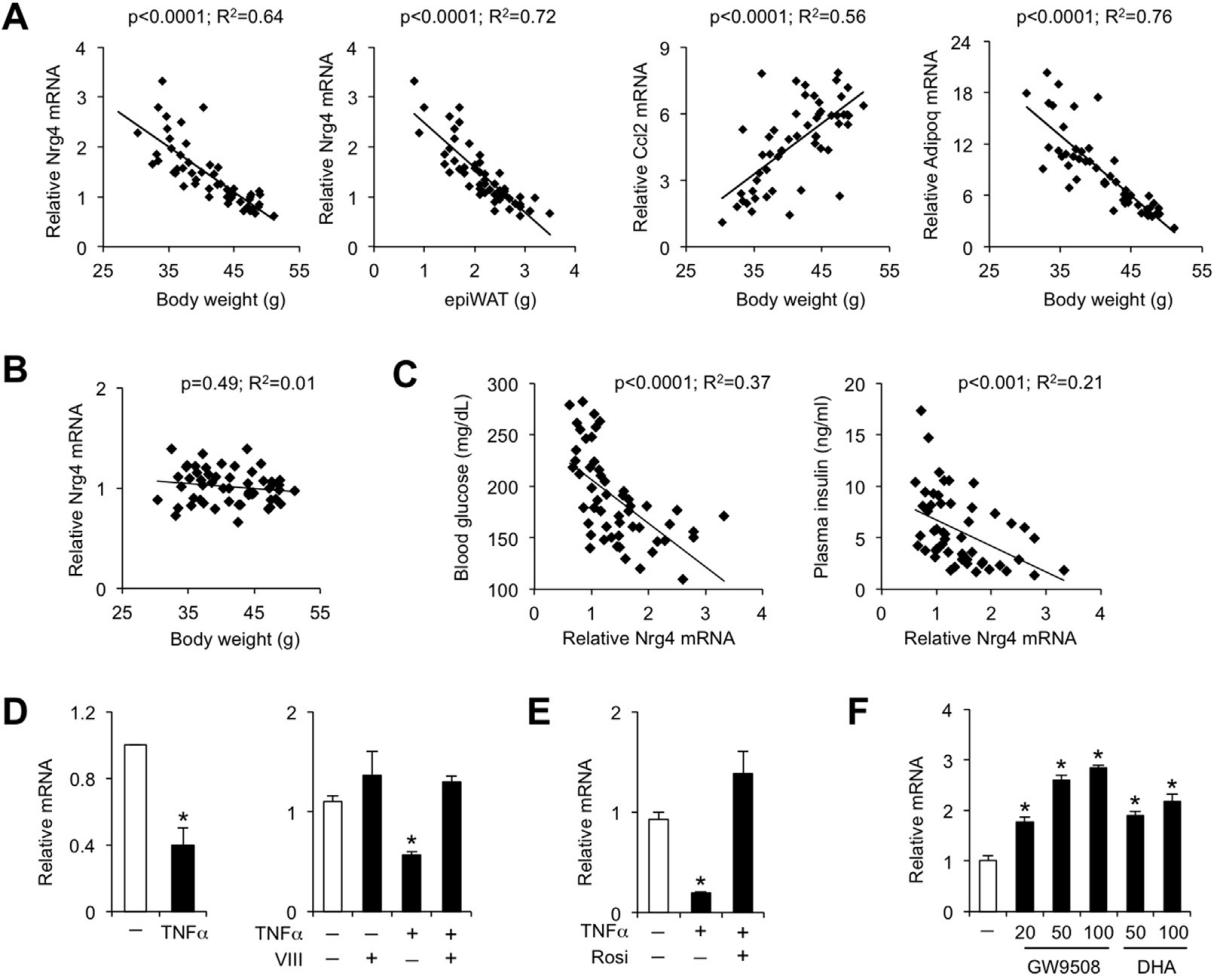
**Regulation of adipose Nrg4 expression**. (A) Correlation of eWAT Nrg4, Ccl2, and Adipoq mRNA levels with body weight and eWAT mass in mice fed HFD for 8 weeks. (B) Correlation of BAT Nrg4 expression with body weight. (C) Correlation of eWAT Nrg4 mRNA levels with blood glucose and plasma insulin concentrations in HFD-fed mice. (D) qPCR analysis of Nrg4 expression in 3T3-L1 adipocytes treated with vehicle (−) or 20 nM TNFα without or with 2 μM inhibitor VIII for 17 h. (E) qPCR analysis of Nrg4 expression in 3T3-L1 adipocytes treated with vehicle or TNFα without or with 10 μM rosiglitazone (Rosi). (F) qPCR analysis of Nrg4 expression in 3T3-L1 adipocytes treated with vehicle, GW9508, or DHA at indicated concentrations (μM) for 17 h. Data represent mean ± sd. *p < 0.01, vs. vehicle; one-way ANOVA.

Nrg4 was identified as a brown fat-enriched endocrine factor that improves obesity-associated insulin resistance and hepatic steatosis [13]. However, the physiological mechanisms underlying the metabolic effects of Nrg4 on glucose and lipid metabolism and energy balance remain incompletely understood. To address this, we first examined adipose tissue expression of Nrg4 in high-fat diet (HFD) induced obesity. Wild type C57BL/6J mice gained various amounts of body weight following two months of high-fat feeding. Nrg4 mRNA levels in epididymal white adipose tissue (eWAT), but not BAT, exhibited a strong inverse correlation with body weight and adiposity (Figure 1A, B). The profile of Nrg4 expression was remarkably similar to that of adiponectin, which has been shown to decrease in eWAT during obesity [1]. On the contrary, the expression of Ccl2, a chemotactic cytokine for macrophages, showed a strong positive correlation with body weight. We further examined the association between eWAT Nrg4 expression and blood glucose and plasma insulin concentrations. As shown in Figure 1C, there was a strong inverse correlation of Nrg4 mRNA levels with blood glucose and plasma insulin concentrations. Mice exhibiting low adipose Nrg4 expression tended to have more severe HFD-induced hyperglycemia and hyperinsulinemia, suggesting that reduced Nrg4 expression may be causally linked to insulin resistance.

Obesity is associated with chronic low-grade inflammation in WAT that is characterized by increased pro-inflammatory cytokine signaling. We next examined how pro-inflammatory and anti-inflammatory stimuli modulate Nrg4 expression in cultured adipocytes. Treatment of differentiated 3T3-L1 adipocytes with TNFα, a prototypical pro-inflammatory cytokine, significantly decreased Nrg4 expression through a mechanism that require NF-kB activation (Figure 1D). Addition of IKK2 inhibitor VIII, a compound that blocks NF-kB activation, abolished the inhibition of Nrg4 expression in response to TNFα. The inhibitory effect of TNFα on Nrg4 expression was also reversed by rosiglitazone (Figure 1E), which exerts powerful anti-inflammatory activities through activation of the nuclear receptor PPARγ [21]. G-protein coupled receptor 120 (GPR120) was recently identified as a receptor for fatty acid ligands, particularly the unsaturated fatty acids such as docosahexaenoic acid (DHA) [22], [23]. Interestingly, treatment of 3T3-L1 adipocytes with DHA or the synthetic GPR120 agonist GW9508 resulted in robust induction of Nrg4 expression (Figure 1F). These results indicate that pro-inflammatory and anti-inflammatory signals exert opposite effects on Nrg4 expression in adipocytes. Further, adipose tissue inflammation is likely a driving force in obesity-associated decrease of Nrg4 expression in adipose tissue.

### 3.2 Nrg4 protects mice from diet-induced obesity by increasing energy expenditure

**Figure 2:**
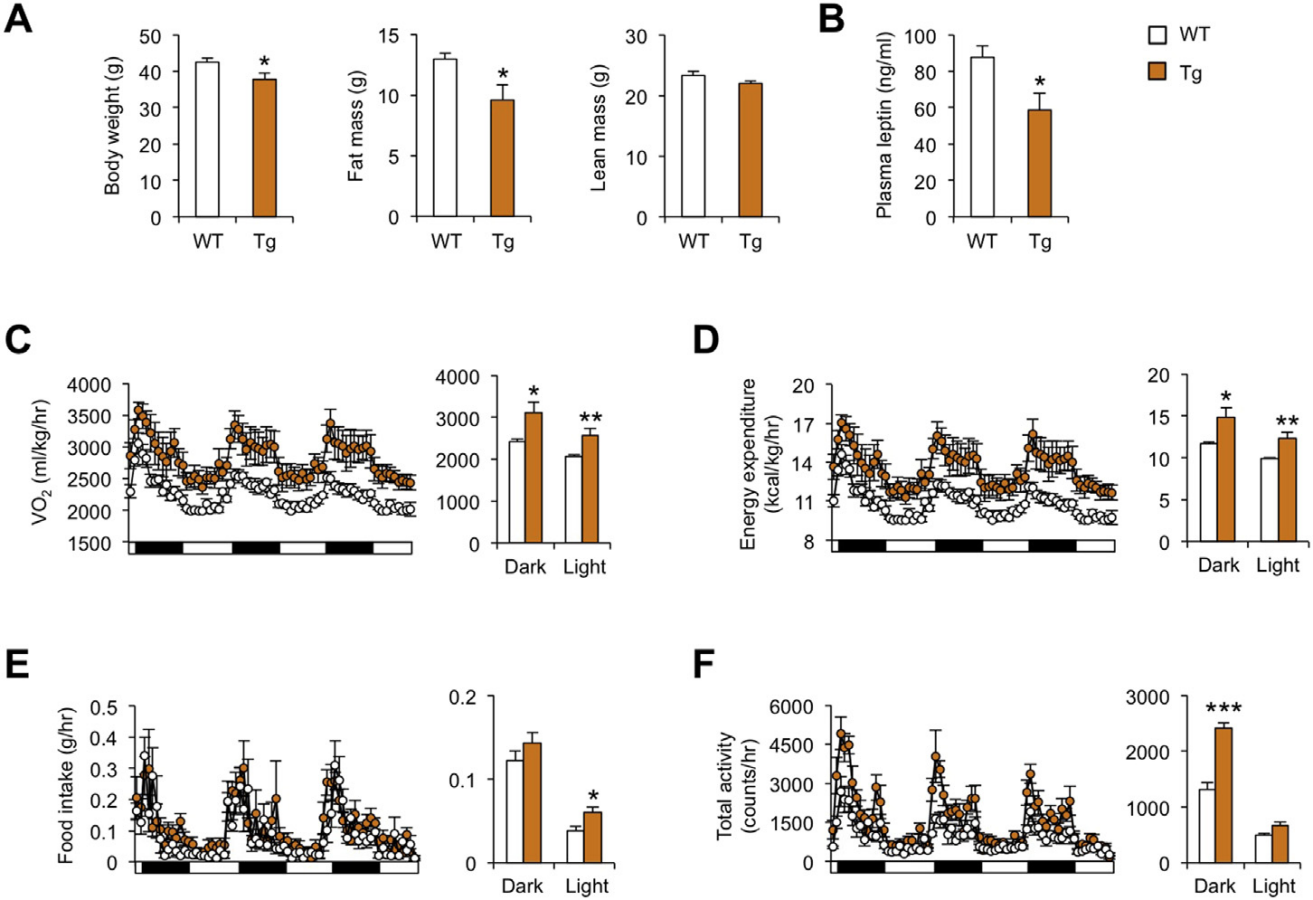
**Body composition analysis and metabolic cage study**. (A) Body weight, fat mass, and lean mass in WT (open, n = 8) and Nrg4 Tg (brown, n = 7) mice fed HFD for 7 weeks. (B) Plasma leptin concentrations. (C) Oxygen consumption rate. Averaged oxygen consumption rate in dark and light phases is indicated on the right. (D) Energy expenditure. (E) Food intake. (F) Total activity counts. Data represent mean ± sem. *p < 0.05, **p < 0.01, ***p < 0.001, WT vs. Tg; two-tailed unpaired Student's t-test.

We previously demonstrated that fat-specific transgenic expression of Nrg4 in adipose tissue ameliorates diet-induced obesity and metabolic disorders [13]. Body composition analysis indicated that, compared to wild type (WT) littermate controls, Nrg4 transgenic (Tg) mice had significantly less fat mass following seven weeks of high-fat feeding (Figure 2A). Whole body lean mass appeared similar between two groups. Plasma leptin levels were lower in Tg mice than control, suggesting that leptin sensitivity is improved in Tg group (Figure 2B). To assess how Nrg4 affects diet-induced weight gain, we performed metabolic cage studies in HFD-fed control and transgenic mice using Comprehensive Laboratory Animal Monitoring System (CLAMS) to assess energy balance. Oxygen consumption rate and energy expenditure were significantly elevated in Nrg4 transgenic mice during both dark and light phases (Figure 2C, D). While food intake was similar in the dark phase, it was moderately higher in Tg mice during the light phase (Figure 2E). Transgenic mice also exhibited increased locomotor activity during the dark phase (Figure 2F). These results strongly suggest that transgenic elevation of Nrg4 levels protects mice from diet-induced obesity by stimulating fuel oxidation and increasing energy expenditure.

### 3.3 Nrg4 improves insulin sensitivity by enhancing peripheral glucose metabolism

**Figure 3:**
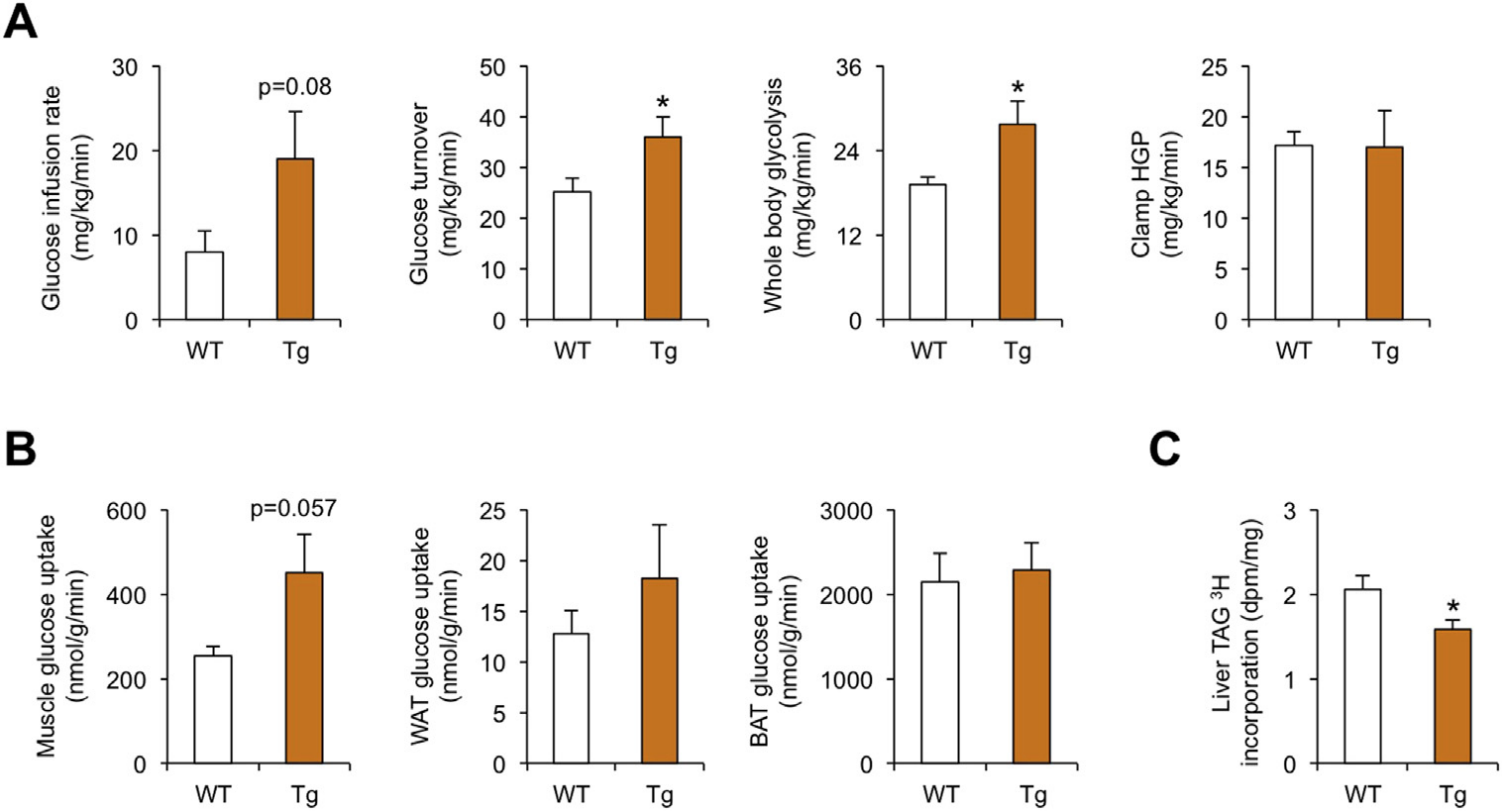
**Hyperinsulinemic-euglycemic clamp study**. (A) Glucose infusion rate, whole body glucose turnover, whole body glycolysis, and hepatic glucose production during clamp in WT (open, n = 9) and Tg (brown, n = 8) mice fed HFD for ten weeks. (B) Insulin-stimulated glucose uptake in skeletal muscle, WAT, and BAT. (C) Incorporation of ^3^H-labeled glucose into liver lipids. Data represent mean ± sem. *p < 0.05, WT vs. Tg; two-tailed unpaired Student's t-test.

We next determined how Nrg4 regulates whole body glucose metabolism using hyperinsulinemic-euglycemic clamp in awake mice after ten weeks of high-fat feeding. Consistent with improved glucose homeostasis, Nrg4 transgenic mice required a higher glucose infusion rate to maintain euglycemia during clamps as compared to WT mice (Figure 3A). Whole body glucose turnover and glycolysis were significantly increased in Tg mice, whereas clamp hepatic glucose production did not differ between Tg and WT mice (Figure 3A). These data indicate that higher glucose infusion rate in Tg mice is largely due to increased glucose metabolism in peripheral organs, but not in liver. Measurements of glucose uptake in individual tissues confirmed that insulin-stimulated glucose uptake in skeletal muscle was enhanced in Tg mice (Figure 3B). In contrast, glucose uptake in white fat and brown fat remained largely unaltered by transgenic expression of Nrg4 in adipocytes. Previous studies demonstrated that Nrg4/ErbB4 signaling attenuates induction of hepatic lipogenesis in response to HFD feeding [13]. Consistent with this, measurement of ^3^H-glucose incorporation in liver triglycerides (TAG) revealed that hepatic lipogenic activity was significantly reduced in Nrg4 transgenic mice (Figure 3C). These results illustrate that Nrg4 exerts a pleiotropic effect on pathways contributing to systemic glucose homeostasis. Increased glucose uptake and utilization likely plays a dominant role in whole body glucose metabolism despite reduced flux of carbohydrates through hepatic lipogenic pathway.

### 3.4 Nrg4 induces a catabolic metabolic state in the liver

**Figure 4:**
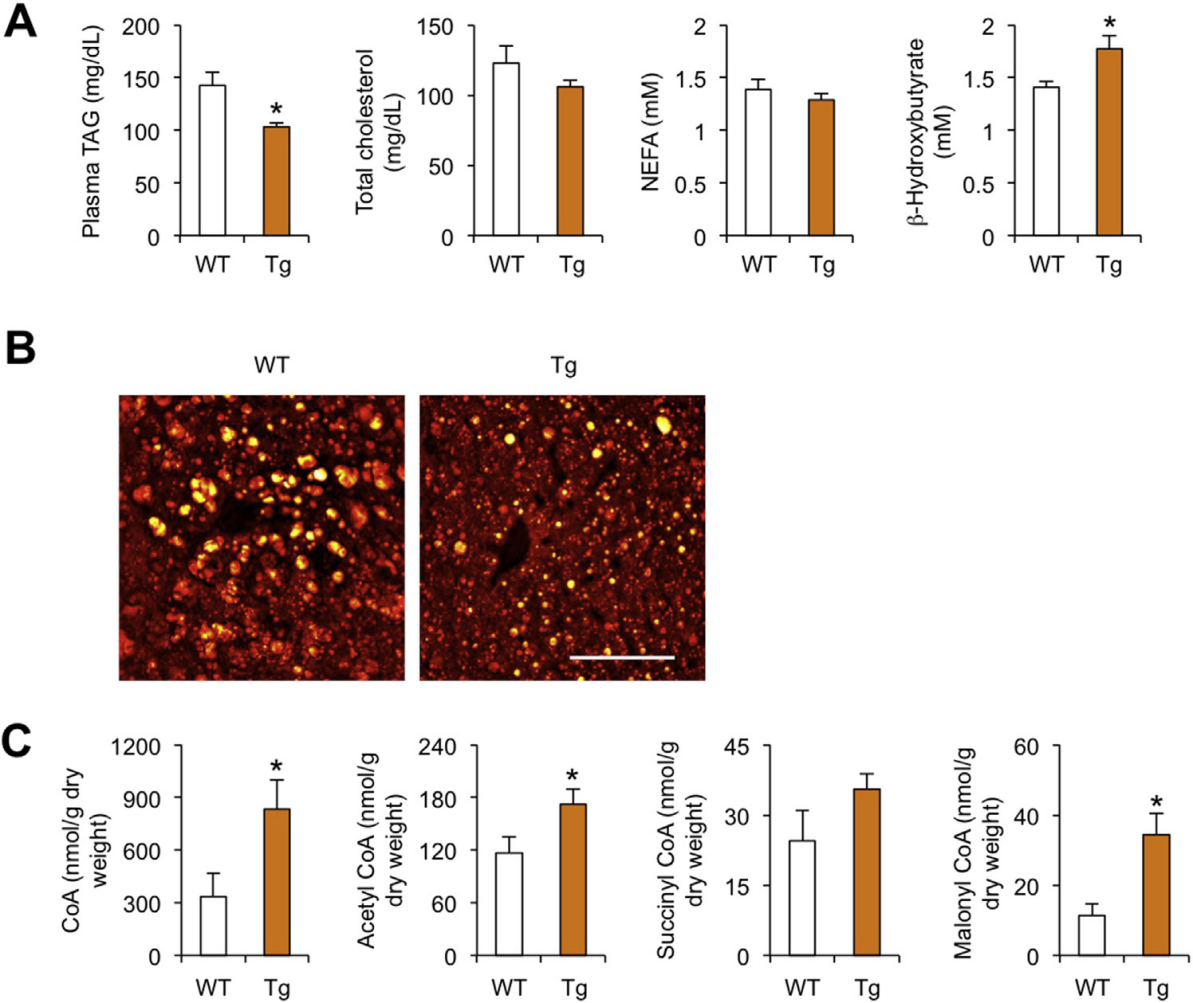
**Effects of Nrg4 transgenic expression on lipid metabolism**. (A) Plasma lipid concentrations in HFD-fed WT (open, n = 10) and Tg (brown, n = 9) mice following overnight starvation. (B) SRS imaging of liver sections. Scale bar = 50 μm. (C) Concentration of CoA metabolites in the liver. Data represent mean ± sem. *p < 0.05, WT vs. Tg; two-tailed unpaired Student's t-test.

Nrg4 transgenic mice developed less severe hepatic steatosis than control following high-fat feeding, whereas mice lacking Nrg4 had more pronounced hepatic fat accumulation. This phenotype is at least in part due to the inhibitory effect of Nrg4/ErbB4 signaling on hepatic lipogenesis. However, whether Nrg4 regulates fatty acid oxidation in the liver has not been examined. Interestingly, Nrg4 transgenic mice exhibited a significantly higher concentration of β-hydroxybutyrate in plasma following overnight fasting (Figure 4A), suggesting that Nrg4 may promote hepatic fatty acid oxidation and ketone production during starvation. Plasma TAG concentration was significantly lower in Tg mice than in control mice, while the concentrations of total cholesterol and non-esterified fatty acids (NEFA) were similar between two groups. We performed Stimulated Raman Scattering (SRS) microscopy, a label-free method to visualize intracellular lipid droplets [19], to assess fat accumulation in control and Tg mice following high-fat feeding. We found that hepatocytes from Tg mouse livers had smaller lipid droplets, consistent with ameliorated hepatic steatosis (Figure 4B). In contrast, liver glycogen content was low following overnight fasting and did not exhibit significant difference between two groups (data not shown). Co-enzyme A (CoA) is a coenzyme that plays a central role in lipid metabolism. Measurements of CoA levels indicated that the concentrations of CoA and acetyl-CoA, but not succinyl-CoA, were significantly elevated in Tg liver (Figure 4C), likely contributing to increased flux of fatty acid β-oxidation and ketogenesis. Surprisingly, malonyl-CoA levels were also elevated in Nrg4 Tg livers, likely due to substrate accumulation as a result of reduced flux through the *de novo* lipogenic pathway.

**Figure 5:**
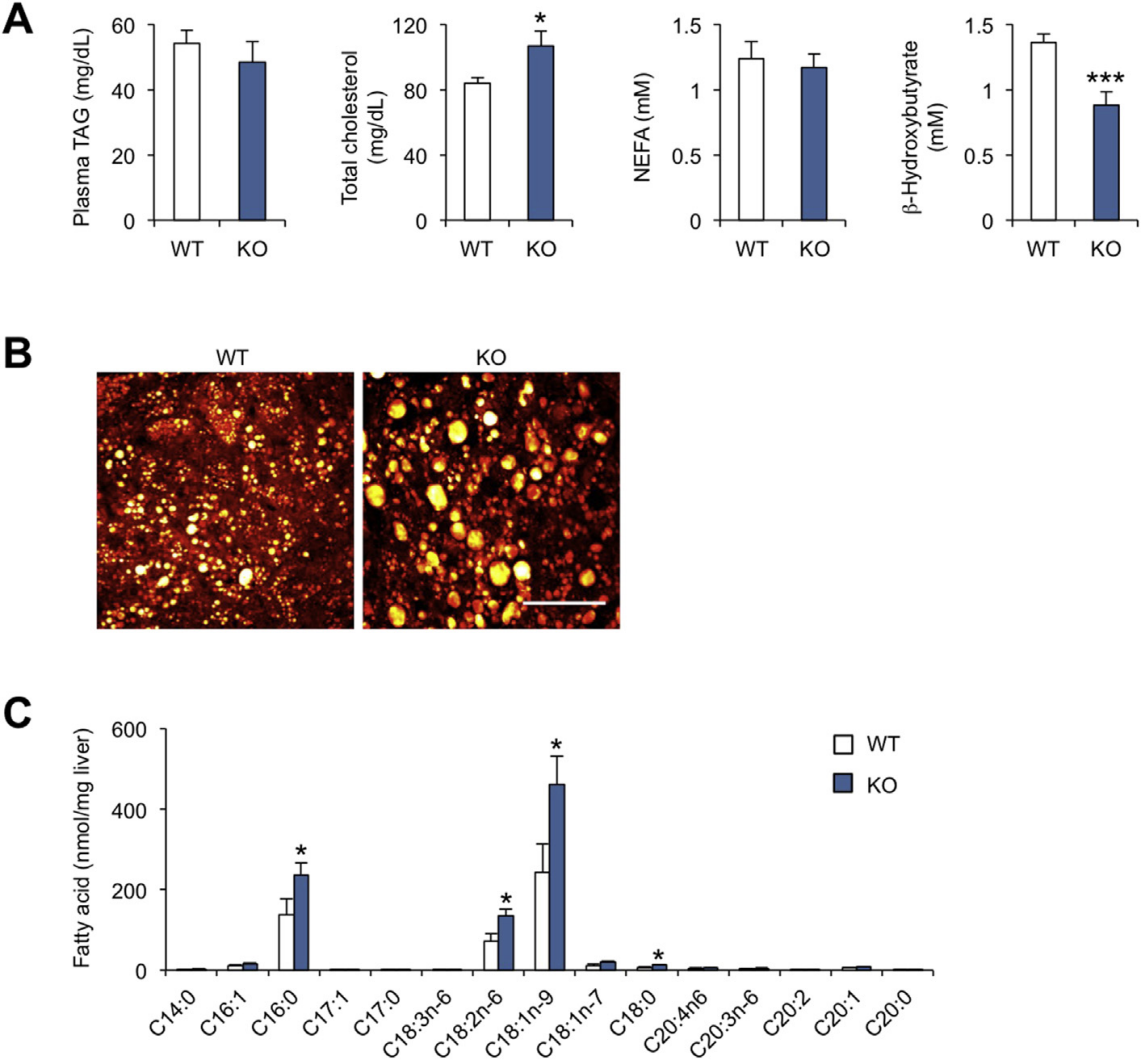
**Effects of Nrg4 deficiency on lipid metabolism**. (A) Plasma lipid concentrations in HFD-fed WT (open, n = 9) and Nrg4 KO (blue, n = 8) mice following overnight starvation. (B) SRS imaging of liver sections. Scale bar = 50 μm. (C) Fatty acid profiling analysis of total liver lipids. Data represent mean ± sem. *p < 0.05, ***p < 0.001, WT vs. KO; two-tailed unpaired Student's t-test.

We next determined whether Nrg4 deficiency impairs hepatic fatty acid oxidation in the fasting state. Consistent with transgenic studies, plasma concentration of β-hydroxybutyrate was significantly lower in Nrg4 knockout (KO) mice following overnight starvation (Figure 5A). While plasma TAG and NEFA levels remained similar, total cholesterol level was moderately elevated in KO group. The differential effects of Nrg4 transgenic overexpression and deficiency on plasma TAG and cholesterol levels suggest that distinct compensatory mechanisms may be activated in gain- and loss-of-function mouse models. SRS microscopy revealed that Nrg4 KO mice developed more severe hepatic steatosis following high-fat feeding, as shown by the presence of large lipid droplets in hepatocytes (Figure 5B). Accordingly, fatty acid profiling of liver lipids indicated that the levels of major fatty acid species, including palmitic acid (C16:0), linoleic acid (C18:2n-6), and oleic acid (C18:1n-9), were significantly elevated in Nrg4 KO mouse liver (Figure 5C). These results suggest that Nrg4 may exert its effects on hepatic fat content by reducing lipogenesis and augmenting fatty acid oxidation.

### 3.5 Regulation of adipose tissue gene expression by Nrg4

**Figure 6:**
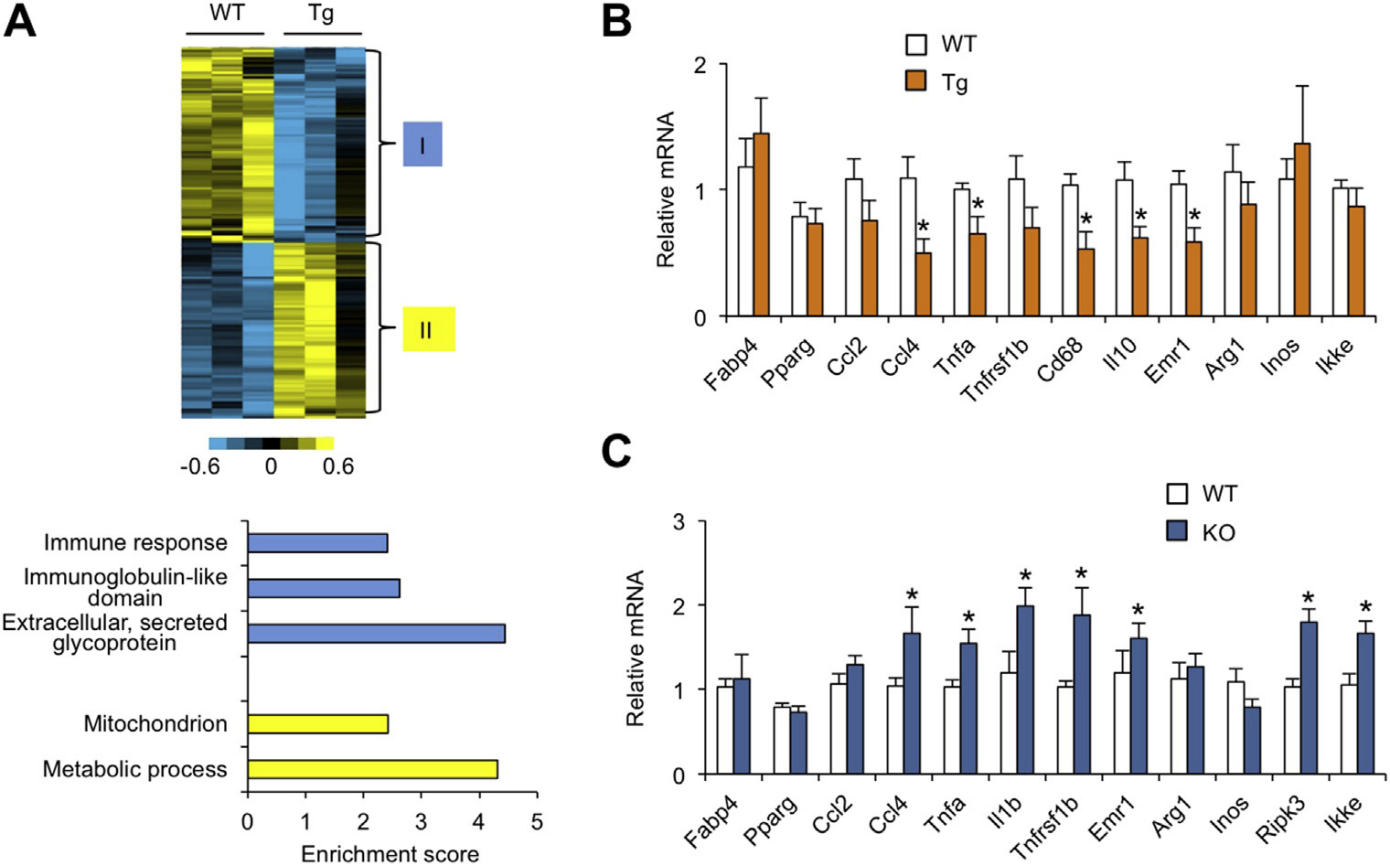
**Regulation of eWAT gene expression by Nrg4**. (A) Two clusters of genes downregulated (I) and upregulated (II) by over 1.6-fold in eWAT from Nrg4 Tg mice (top). Enrichment scores of biological processes for the genes in two clusters (bottom). (B) qPCR analysis of eWAT gene expression in HFD-fed WT (open, n = 10) and Nrg4 transgenic (brown, n = 9) mice. (C) qPCR analysis of eWAT gene expression in HFD-fed WT (open, n = 9) and Nrg4 KO (blue, n = 8) mice. Data represent mean ± sem. *p < 0.05, vs. WT; two-tailed unpaired Student's t-test.

White fat plays a central role in the regulation of fuel storage and also serves as an important source of endocrine hormones. In addition, adipose tissue inflammation has been causally linked to obesity-associated metabolic disorders. Previous Nrg4 binding studies were inconclusive for adipose tissue due to poor preservation of histology on frozen fat sections [13]. Despite abundant Nrg4 expression in adipose tissue, it remains unknown whether Nrg4 may directly or indirectly regulate adipose tissue function. To test this, we performed gene profiling in eWAT from HFD-fed WT and Tg mice using microarray. We identified two clusters of genes that exhibit significant downregulation (81 genes) or upregulation (73 genes) in response to transgenic expression of Nrg4 (Figure 6A and Supplementary Table S1). Pathway analysis of these two clusters indicated that genes downregulated by Nrg4 were enriched for those involved in immune and inflammatory response. Genes encoding proteins predicted to contain immunoglobulin-like domain and secreted glycoproteins were also enriched in this cluster. The upregulated cluster was enriched for genes involved in mitochondrial function and energy metabolism. QPCR analysis indicated that the expression of several genes involved in inflammatory signaling, including Ccl4, Tnfα, Cd68, Il10, and Emr1, was significantly lower in transgenic eWAT (Figure 6B). On the contrary, mRNA expression for inflammation-associated genes was elevated in eWAT from HFD-fed Nrg4 KO mice (Figure 6C). The expression of adipogenic genes (Pparγ, Fabp4) remained similar. These results are consistent with recent studies that demonstrate anti-inflammatory activities of Nrg4/ErbB4 signaling in adipose tissue and colon [24], [25].

### 3.6 Nrg4 promotes a healthy profile of adipokine secretion

**Figure 7:**
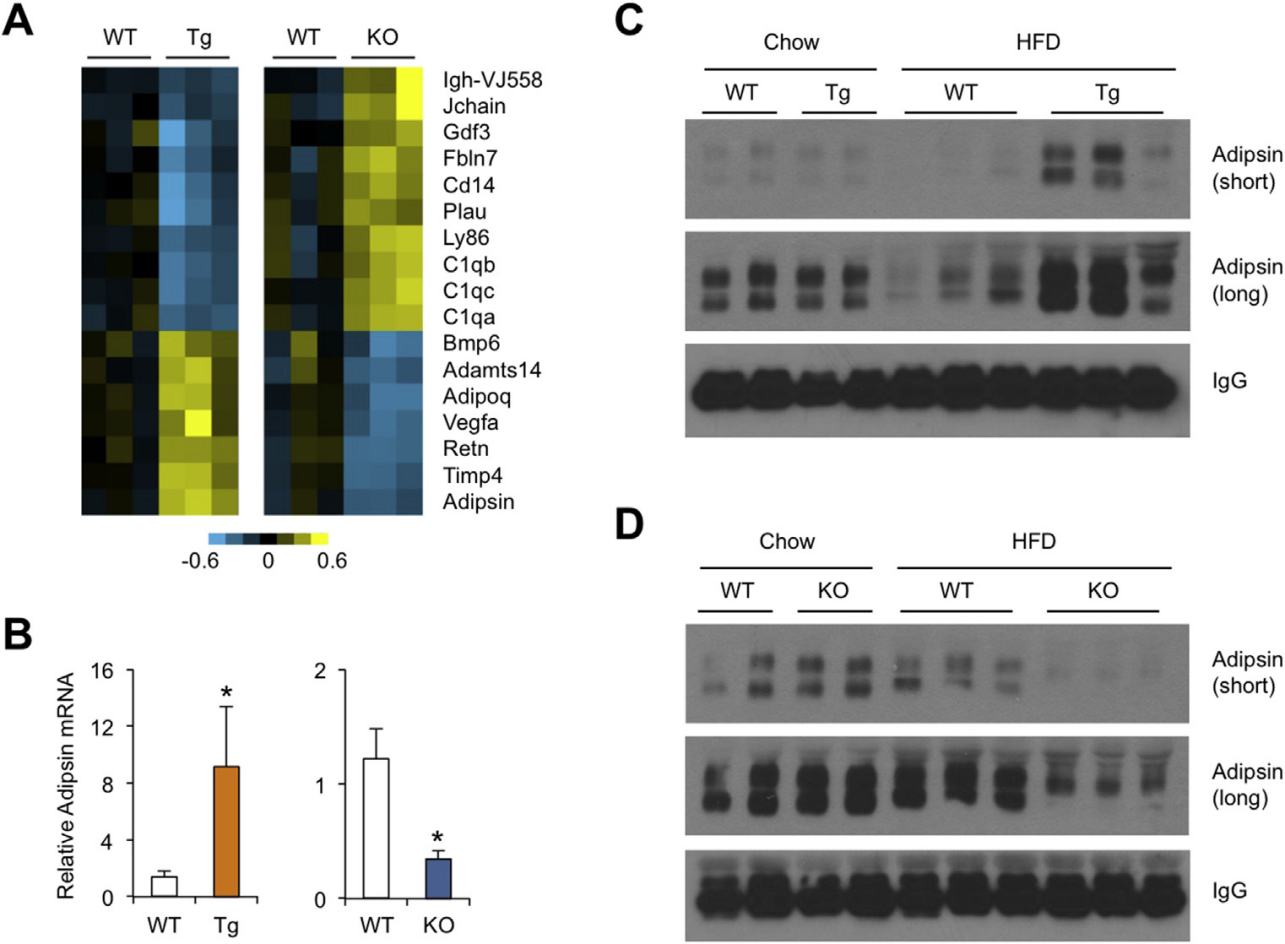
**Regulation of eWAT secretome by Nrg4**. (A) Reciprocal regulation of secreted factor gene expression in eWAT from Nrg4 transgenic and knockout mice. Shown are genes that exhibit an increase or decrease in expression by over 1.4-fold based on microarray expression values. (B) qPCR analyses of adipsin gene expression in eWAT from a cohort of HFD-fed WT (open, n = 10) and Nrg4 Tg (brown, n = 9) mice and a cohort of WT (open, n = 9) and Nrg4 KO (blue, n = 8) mice. Data represent mean ± sem. *p < 0.05, vs. WT; two-tailed unpaired Student's t-test. (C) Immunoblotting of plasma samples from chow or HFD-fed WT and Tg mice. (D) Immunoblotting of plasma samples from chow or HFD-fed WT and Nrg4 KO mice.

Adipose tissue releases diverse secreted factors that act locally or function as endocrine regulators. To determine whether Nrg4 plays a role in modulating the adipocyte secretome, we examined microarray expression levels of the genes that are included in the mouse secretome list and predicted to encode secreted proteins. We identified a list of genes encoding secreted proteins that exhibited transcriptional regulation in Nrg4 transgenic and knockout mouse eWAT in the opposite manner (Figure 7A). Heat map representation revealed that transgenic expression of Nrg4 increased mRNA expression of several adipokines that have been demonstrated to exert beneficial metabolic effects, including Adipsin (Complement factor d, Cfd), Adiponectin, and Vascular endothelial growth factor a (Vegfa). Adipsin has been shown to improve β cell function in diabetes and its expression is downregulated in obesity [26], [27]. While adiponectin exerts its beneficial effects on diverse tissues including skeletal muscle and the liver [28], [29], VEGFα appears to improve metabolic physiology by acting locally to enhance adipose tissue vascularization and function [30], [31]. The expression of genes involved in the classical complement activation pathway, including C1qa, C1qb, and C1qc, were downregulated in eWAT from Nrg4 transgenic mice. Remarkably, Nrg4 deficiency resulted in elevated expression for these factors. C1qa expression has been shown to be elevated in visceral adipose tissue from obese patients and its deficiency protects mice from HFD-induced insulin resistance [32], [33]. We confirmed the expression of adipsin using qPCR (Figure 7B). Further, while plasma adipsin levels were similar between chow-fed WT and Tg mice, transgenic expression of Nrg4 greatly increased adipsin levels in circulation following high-fat feeding (Figure 7C). Similarly, no difference was observed between WT and KO mice under chow-fed condition. Nrg4 deficiency resulted in lower adipsin levels in diet-induced obese mice, but not chow-fed lean mice (Figure 7D). These results strongly suggest that Nrg4 promotes a healthy adipokine profile that likely contributes to the amelioration of obesity-associated metabolic disorders.

## 4 Discussion

**Figure 8:**
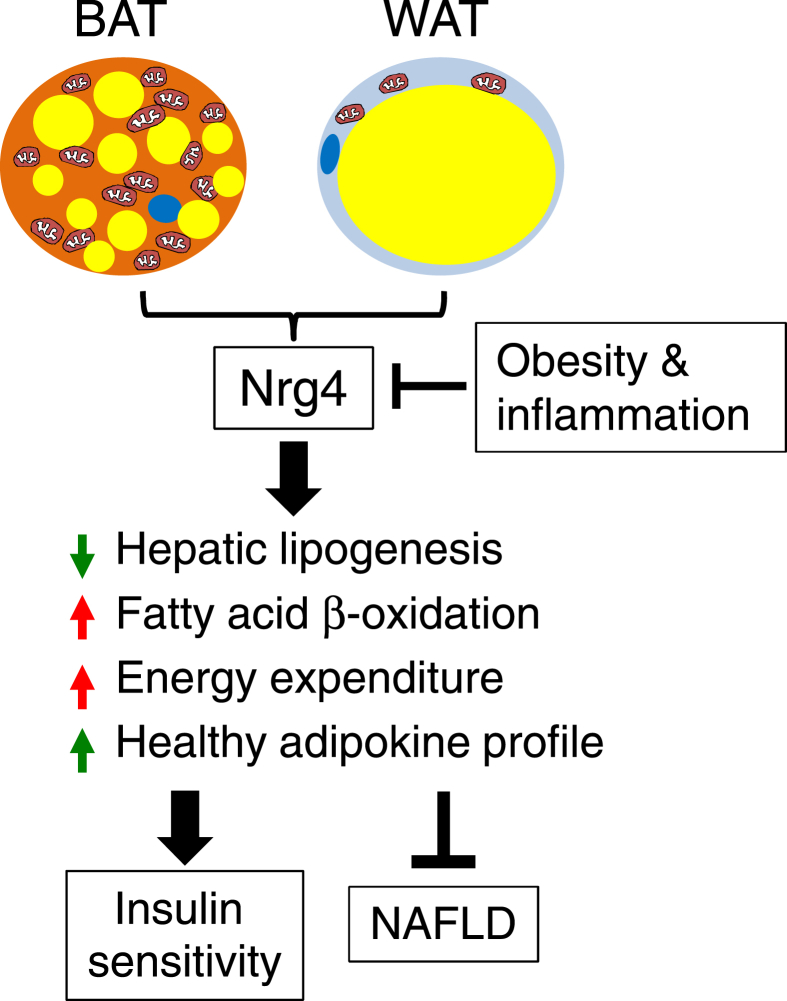
**A model depicting the pleiotropic action of Nrg4 and its role in metabolic homeostasis**. Nrg4 expression is repressed by pro-inflammatory signaling in white adipose tissue. Nrg4 preserves insulin sensitivity and ameliorates NAFLD by attenuating hepatic lipogenesis, augmenting fuel oxidation and energy expenditure, and maintaining a healthy adipokine profile.

Adipose tissue releases a plethora of secreted factors that act locally or on distal tissues to influence metabolic physiology. Nrg4 was recently identified as a brown fat-enriched endocrine factor that ameliorates HFD-induced insulin resistance and hepatic steatosis [13]. Despite these intriguing findings, the physiological mechanisms through which Nrg4 regulates systemic energy balance and glucose and lipid metabolism have not been elucidated. In this study, we provide the novel demonstration that Nrg4 reduces HFD-induced weight gain in mice by enhancing basal metabolic rate and energy expenditure. Transgenic activation of Nrg4 signaling resulted in increased whole body glucose turnover and glycolysis that was in part due to augmented skeletal muscle glucose utilization. We also identified hepatic fatty acid β-oxidation and ketogenesis as a new downstream target of Ngr4 in hepatocytes, likely contributing to the protective effects of Nrg4 on hepatic steatosis. Finally, Nrg4 promotes a beneficial adipokine profile in white fat that was associated with reduced expression of genes involved in inflammatory response. This study illustrates the surprisingly pleiotropic effects of Nrg4 on energy and nutrient metabolism (Figure 8).

Neuregulin proteins belong to a small family of extracellular ligands that includes Nrg1, Nrg2, Nrg3, and Nrg4 [34], [35], [36]. Among these, Nrg1, 2, and 3 are highly expressed in the central nervous system. Nrg4 is the only member that exhibits high levels of expression in brown and white fat. Remarkably, Nrg4 mRNA expression in white adipose tissue inversely correlates with adiposity in both rodents and humans [13]. These observations strongly suggest that defective Nrg4 secretion may lead to a hormonal insufficiency during obesity that exacerbates the progression of metabolic disorders. In support of this, plasma Nrg4 levels were inversely associated with the risk for metabolic syndrome in obese Chinese adults [37]. While adipocyte Nrg4 expression is suppressed by pro-inflammatory cytokines, anti-inflammatory agents such as rosiglitazone and agonists for GPR120 exert an opposite effect. These results illustrate that the balance between pro-inflammatory and anti-inflammatory signals is likely a key driver of adipose tissue Nrg4 expression.

Because Nrg4 binding appears to be restricted to hepatocytes, the pleiotropic effects of Nrg4 on glucose and lipid metabolism came somewhat as a surprise. It is likely that these metabolic effects are due to a combination of direct Nrg4 action and secondary consequences of Nrg4 manipulations. For example, adipose tissue expresses very low levels of ErbB3 and ErbB4, two receptors that mediate Nrg4 signaling. It is possible that the shift in adipokine profile in response to transgenic expression and deficiency of Nrg4 occurs as a result of Nrg4 signaling in other tissues. The molecular nature of such a regulatory circuitry remains unknown.

Previous studies demonstrated that ErbB receptors are expressed in skeletal myocytes and that a fragment corresponding to the EGF-like domain of Nrg1 was capable of stimulating glucose uptake in cultured C2C12 myotubes [38], [39], [40]. Nrg1 treatment improved whole body glucose metabolism in db/db mice and diabetic rats [41], [42]. Whether Nrg4 may act directly on skeletal muscle to promote glucose uptake and utilization remains an important question to be addressed in future studies. Beyond peripheral tissues, ErbB4 is highly expressed in the central nervous system, including the hypothalamus [43], [44], [45]. Importantly, ErbB4 polymorphism was recently shown to strongly associate with body mass index in a genome-wide association study [46]. Because Nrg4 transgenic mice exhibited reduced fat mass and elevated energy expenditure, our findings raise the possibility that Nrg4/ErbB4 signaling may be a genetic factor for human obesity and its associated metabolic disorders. Given the pleiotropic metabolic benefits elicited by Nrg4, biologic therapeutics targeting this pathway may provide an effective treatment that simultaneously targets type 2 diabetes and NAFLD in patients.
